# Deodorant use and related adverse effects: A cross-sectional survey among Palestinian students

**DOI:** 10.4314/ahs.v24i1.34

**Published:** 2024-03

**Authors:** Iyad Ali, Naser Shraim, Anwar Younes, Reem Sawafta, Asma Sbeih, Samar Khater

**Affiliations:** 1 Department of Biochemistry, Faculty of Medicine and Health Sciences, An-Najah National University, Nablus, Palestine; 2 Department of Pharmacy, Faculty of Medicine and Health Sciences, An-Najah National University, Nablus, Palestine; 3 Faculty of Pharmacy, Applied Science University, Amman, Jordan

**Keywords:** deodorants, antiperspirants, adverse effects, skin, students, physician consultation

## Abstract

**Background:**

Deodorants are widely used to mask unpleasant body odors. They are reported to cause some adverse effects depending on the form and ingredients. The purpose of this study was to assess the prevalence of deodorant use and related adverse effects among Palestinian students.

**Methods:**

This cross-sectional study was carried out at An-Najah National University from Aug 2018 to Mar 2020. A questionnaire was constructed using a Google survey form. All analyses were done using SPSS 21.0, Fisher test was used for comparative analysis and the P-value < 0.05 was considered as statistically significant.

**Results:**

A total of 554 students participated in the study. About 93% of the participants were using deodorants and adverse effects were reported by 25% of the participants. The reported adverse effects were itching (26%), coloration (25%), sneezing (21%), and eye redness (8%). Only 4.1% of the participants visited a physician for counselling regarding various adverse effects.

**Conclusion:**

Deodorants were widely used by Palestinian students with reported adverse effects, but these side effects rarely prompted the participants to seek medical advice.

## Introduction

Deodorants are pharmaceutical preparations which exhibit antimicrobial activity and mask perspiration odor 1. Some deodorants are derived from natural sources, while many are synthetic products. Besides, these products typically contain perfume and mixtures of various chemical compounds 2. A subclass of deodorants contains antiperspirants that block the sweat glands through the action of astringent salts, such as aluminium and zinc 3.

Deodorants are manufactured in many forms like gels, powders, sprays, sticks and rolls, to give the consumer the choice to choose a suitable product 4. In the United States (U.S) deodorants are classified and regulated as cosmetics by the U.S. Food and Drug Administration (FDA) 5 and have become one of the most profitable industries in the nation's personal-care business 6.

The daily use of deodorants has become everybody's habit, especially among young females 7, as women spend more money than men for products related to their personal care 8. However, deodorants of both sexes contain similar active ingredients at similar concentrations, but may differ in their fragrance content based upon cultural norms and personal preferences 9.

Despite the advantages of deodorants use, some disadvantages were reported after being used over a long time 10. Some deodorants contain many harmful components like aluminium, which is present in most deodorants, that can interact with the DNA and cause the cell to become cancerous 11. A recent study suggested that the frequent use of antiperspirants can cause aluminium to accumulate in breast tissue, nevertheless this doesn't prove that aluminium salts can cause breast cancer 4.

Additionally, the perfume or fragrance odor itself may be irritating or offensive to the user's skin, respiratory system and/or olfactory senses 12. Lately, research has proven that many harmful effects of deodorants depend on their forms. Some experts are warning that inhaling chemicals from spray aerosols may cause allergic skin reactions, asthma, and breathing difficulties 13. While axillary granulomas, pruritus, and acute inflammation are associated with the use of stick form 14, another study at Mekelle University, revealed that 97.8% of female students had a habit of using cosmetics, and 18.4% encountered adverse effects due to deodorants use 15. The aim of this study was to assess the prevalence of deodorant use and to explore the related adverse effects among Palestinian students.

## Methods

### Study setting and design

This non-randomized cross-sectional study was conducted at An-Najah National University (ANNU) in Nablus, Palestine. This study was conducted between Aug 2018 and Mar 2020. It is a questionnaire-based study. The questionnaire was written in Arabic, tested by a pilot sample (N=20), and some minor modifications were made to construct the final version. Ethical approval was obtained from the Institutional Review Board at ANNU.

### Population and sampling

The sample size was determined using Raosoft sample size calculator, considering a 5% margin of error, a confidence level of 95% and a response distribution of 50%. The age of the participants was between 18-25 years and they belonged to different academic fields.

### Data collection and analysis

The questionnaire consisted of 27 questions and was placed on a Google Survey service. The questionnaire was published on different students' social groups and close-ended questions were used. A brief description about the study and its objectives was placed at the beginning of the questionnaire. The questionnaire was divided into three parts, the first part covered the socio-demographic characteristics of the participants, brand name of deodorants, and the knowledge about the antiperspirant content. The second part investigated the duration of use, frequency of daily use, deodorant form, and the application method. The last part covered the potential adverse effects caused by deodorants in addition to medical consultation. All results were analysed by using SPSS statistics program (SPSS Inc., Chicago, IL, USA), version 21.0. Fisher test was used for comparative analysis and p-value < 0.05 was accepted as statistically significant.

## Results

Five hundred and fifty-seven participants answered the questionnaire and 554 completed the survey. The majority of the participants (79%) were female, and all students were in the age group of 18-25 years distributed on different academic years (Table 1). Participants were equally residing in cities (49%) and villages (48%). About two-thirds of the participants possessed knowledge about the antiperspirants content of deodorant, and 57% had a habit of reading the information printed on deodorant products. The majority of the participants (93%) were using deodorants, and half of them had a habit of changing their deodorant products frequently for different reasons including, odor (56%), poor stability (18%) and cost (11%) (Table 1). About 43% of participants used deodorant products daily and buy these products from supermarkets (44%), pharmacies (32%), and from road side sellers (2%).

**Table 1:** Demographic and deodorant utilization pattern

Variables			No. (%)
**Gender**		Male	119 (21)
		Female	435 (79)
**Place of living**		City	272 (49)
		Village	266 (48)
		Camp	16 (3)
**Antiperspirant Knowledge**			
		Yes	343 (62)
		No	211 (38)
**Antiperspirant content**			
		Yes	316 (57)
		No	238 (43)
**Deodorant use**		Yes	515 (93)
		No	39 (7)
**Adverse effects**		Yes	128 (25)
		No	387 (75)
**Changing deodorant**			
		Yes	258 (50)
		No	257 (50)
**Why changing deodorant**			
		Odor	144 (56)
		Poor stability	46 (18)
		Cost	29 (11)
		Granulomatous	15 (6)
		Coloration	12 (4)
		Other	12 (4)
**Frequency of use**			
		Once daily	221 (43)
		Twice daily	124 (24)
		>twice daily	82 (16)
		After shower	62 (12)
		Irregular	26 (5)
**Source of deodorant**			
		Supermarkets	227 (44)
		Pharmacies	167 (32)
		Street sellers	8 (2)
		Not specific	113 (22)

Approximately 25% of participants experienced at least one adverse effect. These adverse effects included skin coloration (25%), skin itching (26%), sneezing and nasal congestion (21%), and eye redness (8%). Only 4% of the participants consulted a health professional after developing different adverse effects (Table 2).

**Table 2:** Adverse effects of deodorant and doctor consultation

Adverse effects		No. (%)
**Coloration**	No	387 (75)
	Yes	128 (25)
	Black	79 (62)
	Red	46 (36)
	Blue	3 (2)
**Itching**	Yes	134 (26)
	No	381 (74)
**Sneezing & Congestion**	Yes	108 (21)
	No	407 (79)
**Eye redness**	Yes	41 (8)
	No	474 (92)
**Consulting a doctor**	Yes	21 (4)
	No	494 (96)

The adverse effects of deodorants were insignificantly associated with gender (Table 3). Coloration was reported by 14% of males and 28% of females (p value <0.001), itching was reported by 25% of males and 26% of females (p value = 0.314), sneezing and congestion was reported by 23% of males and 20% of females (p value =0.165), eye redness was reported by 11% of males and 7% of females (p value =0.174). While, seeking medical advice was neglected by both sexes (5% males and 4 % females).

**Table 3:** Comparison of deodorant adverse effects between males and females

Characteristics		Male (%)	Female (%)	Total (%)	P-value
**Coloration**	Yes	15 (14)	113 (28)	128 (25)	<0.001
	No	96 (86)	291 (72)	387 (75)	
**Itching**	Yes	28 (25)	106 (26)	134 (26)	0.314
	No	83 (75)	298 (74)	381 (74)	
**Sneezing & Congestion**	Yes	26 (23)	82 (20)	108 (21)	0.165
	No	85 (77)	322 (80)	407 (79)	
**Eye redness**	Yes	12 (11)	29 (7)	41 (8)	0.174
	No	99 (89)	375 (93)	474 (92)	
**Consulting a doctor**	Yes	6 (5)	15 (4)	21 (4)	0.085
	No	105 (95)	389 (96)	494 (96)	

Inquiring about the duration of deodorant usage revealed that a majority of the participants, specifically 60%, had been utilizing deodorants for over three years. Participants across all usage durations have reported different adverse effects (Table 4). Out of the total participants, 41 reported experiencing eye redness as a negative effect. Among them, 14% had been using deodorant for less than a year, 9% had been using it for 1-3 years, and 6% had been using it for more than three years. No significant correlation was found between other adverse effects and the duration of deodorant use. New users where more often to seek medical consultation (p value=0.001) compared with the old users.

**Table 4:** Deodorant adverse effects based on the duration of use

Characteristics		<1 year No. (%)	1-3 years No. (%)	>3 years No. (%)	Total No. (%)	P-value
**Coloration**	Yes	12 (17)	28 (20)	88 (29)	128 (25)	0.625
	No	58 (83)	110 (80)	219 (71)	387 (75)	
**Itching**	Yes	29 (42)	41 (30)	64 (21)	134 (26)	0.056
	No	41 (58)	97 (70)	243 (79)	381 (74)	
**Sneezing & Congestion**	Yes	17 (24)	37 (27)	54 (18)	108 (21)	0.120
	No	53 (76)	101 (73)	253 (82)	407(79)	
**Eye redness**	Yes	10 (14)	13(9)	18 (6)	41 (8)	0.033
	No	60 (86)	125 (91)	289 (94)	474 (92)	
**Consulting a doctor**	Yes	9 (13)	4 (3)	8 (3)	21 (4)	0.001
	No	61(87)	127 (97)	306 (97)	494 (96)	

The order of the commonly used deodorant forms were spray, roll, gel, powder, and stick. Among the powder users (45 participants), 22% reported skin coloration, 53% reported skin itching, 27% reported sneezing and congestion, and 13% reported eye redness. About 18% of powder users consulted a health professional (Table 5). Among the 265 spray users, 21% reported skin coloration, 37% reported skin itching, 28% reported sneezing and congestion, and 11% reported eye redness. While among the gel users, 27% reported skin coloration, 17% reported skin itching, 10% reported sneezing and congestion, 5% reported eye redness, and only 5% consulted a health professional. Regarding the roll users (124 participants), 27% reported skin coloration, 2% reported skin itching, 11% reported sneezing and congestion, 2% reported eye redness, and only 2% consulted a health professional. None of stick users reported skin itching, eye redness, but 68% reported skin coloration, 5% reported sneezing and congestion, and 5% consulted a health professional.

**Table 5:** Deodorant adverse effects based on deodorant type

Characteristics		Powder No. (%)	Spray No. (%)	Gel No. (%)	Roll No. (%)	Stick No. (%)	Total No. (%)	P-value
**Coloration**	Yes	10 (22)	54 (21)	16 (27)	33 (27)	15 (68)	128 (25)	0.072
	No	35 (78)	211 (79)	43 (73)	91 (73)	7 (32)	387 (75)	
**Itching**	Yes	24 (53)	97 (37)	10 (17)	3(2)	0 (0)	134 (26)	
	No	21 (47)	168 (63)	49 (83)	121 (98)	22 (100)	381 (74)	0.001
**Sneezing & Congestion**	Yes	12 (27)	75 (28)	6 (10)	14 (11)	1 (5)	108 (21)	0.001
	No	33 (73)	190 (72)	53 (90)	110 (89)	21 (95)	407 (79)	
**Eye redness**	Yes	6 (13)	30 (11)	3 (5)	2 (2)	0 (0)	41(8)	0.005
	No	39 (87)	235 (89)	56 (95)	122 (98)	22(100)	474(92)	
**Consulting doctor**	Yes	8 (18)	6 (2)	3 (5)	3 (2)	1 (5)	21 (4)	0.001
	No	37 (82)	259 (98)	56 (95)	121(98)	21 (95)	494 (96)	

## Discussion

The prevalence of deodorants use among university students was 93%, which was higher than what was reported by others 16. This might be explained by the age differences since the participants in this study were youth (18-23 years) and keener to maintain good body odor. Female participants, as expected, were using deodorants more than males, which is in agreement with other studies 17, 18. Moreover, participants tended to change their deodorant due to deodorant odor rather than adverse effects.

A previous study showed that among different cosmetics, deodorants received most of the reported complaints 9. The presence of the antiperspirant label on deodorant products has piqued the interest of consumers, as more than half of the participants (57%) reported reading about it. Antiperspirant's astringent salts such as aluminium have been reported to be absorbed through the armpit skin 19 and are related to some diseases like Alzheimer's disease and breast cancer 20-22 and this may hinder the use of deodorants by some people.

Males have experienced more adverse effects than females, in spite of the similar composition of the deodorants for both sexes except for the fragrance 9, but this finding was statistically insignificant in our study. Usually, the fragrance may irritate the user's skin, respiratory system, and olfactory senses 12. According to the present study, individuals who were new users of deodorant reported a higher incidence of adverse reactions and more frequent visits to physicians compared to those who had been using it for a longer period of time. This indicates that the adverse effects associated with deodorant use might be reduced with time. A study from Denmark also showed that individuals who use deodorant for a longer time may tolerate the allergen in the deodorant depending on several factors 23. In contrast, another study showed that adverse reactions/span>were more dominant at long-term use 24.

The deodorant's adverse effects usually depend on the deodorant's form, for example, the presence of talcum material in the powder form, which is irritating to the respiratory tract and considered carcinogenic 25. The spray form was the predominant cause of itching and sneezing due to its volatile nature, which can be easily inhaled into the respiratory system causing irritation. In addition, the spray users did not follow the instructions like, not to use the spray in closed places, or using it from a distance less than 15 cm. Moreover, some users may not shake the bottle before using it, which causes accumulation of salts at the bottom and alcohol content of the spray may dry the skin and cause irritation (Table 5). A study published in the British journal of dermatology has shown that axillary granulomas, pruritus and acute inflammation were associated with the use of stick form 14. In our study the stick form appeared to cause the maximum skin coloration but the minimum itching, sneezing and eye redness, and this could be partially due to lower alcohol content and minimum spreading ability. A significant association was found between deodorants forms and itching (p-value= 0.001), sneezing (p-value= 0.001), eye redness (p-value= 0.005) but not with coloration (p-value=0.072). Finally, only 4% of the participants were concerned about the development of adverse effects and consulted a health professional and this is lower than other studies 26.

## Strength and limitations

This is the first study in Palestine to assess the prevalence of deodorants use and associated adverse effects. A limitation of this study could arise from the fact that it was a web-based survey.

## Conclusion

Deodorants have become everybody's daily use habit, especially among young people. Different adverse effects were correlated with the form of deodorant used and with the duration of use. Many participants suffered from adverse effects with few participants consulted a health professional regarding these adverse effects.

## Data Availability

The authors confirm that the data supporting the results of this study are available in the article.
